# Plakofuranolactone as a Quorum Quenching Agent from the Indonesian Sponge *Plakortis* cf. *lita*

**DOI:** 10.3390/md15030059

**Published:** 2017-02-28

**Authors:** Valeria Costantino, Gerardo Della Sala, Kumar Saurav, Roberta Teta, Rinat Bar-Shalom, Alfonso Mangoni, Laura Steindler

**Affiliations:** 1 The Blue Chemistry Lab Group, Department of Pharmacy, University of Naples Federico II, 80131 Napoli, Italy; gerardo.dellasala@unina.it (G.D.S.); roberta.teta@unina.it (R.T.); alfonso.mangoni@unina.it (A.M.); 2 Department of Marine Biology, Leon H. Charney School of Marine Sciences, University of Haifa, Mt. Carmel, 31905 Haifa, Israel; sauravverma17@gmail.com (K.S.); rbar-shal@univ.haifa.ac.il (R.B.-S.); lsteindler@univ.haifa.ac.il (L.S.)

**Keywords:** antibacterial from marine sponges, antivirulence lead compounds, quorum sensing system, plakofuranolactone, quorum quenching activity, marine sponges, *Plakortis lita*, *Pseudomonas aeruginosa*, reporter gene assays

## Abstract

There is an urgent need for novel strategies to fight drug resistance and multi-drug resistance. As an alternative to the classic antibiotic therapy, attenuation of the bacteria virulence affecting their Quorum sensing (QS) system is a promising approach. Quorum sensing (QS) is a genetic regulation system that allows bacteria to communicate with each other and coordinate group behaviors. A new γ-lactone that is capable of inhibiting the LasI/R QS system, plakofuranolactone (**1**), was discovered in the extract of the marine sponge *Plakortis* cf. *lita*, and its structure, including absolute configuration, was determined by NMR spectroscopy, MS spectrometry, and quantum-mechanical prediction of optical rotation. The quorum quenching activity of plakofuranolactone was evaluated using reporter gene assays for long- and short-chain signals (*E. coli* pSB1075, *E. coli* pSB401, and *C. violeaceum* CV026) and was confirmed by measuring the total protease activity (a virulence factor which is under control of the LasI/R system) of the wild-type *P. aeruginosa* PAO1. Further research will be pursued to assess the potential of plakofuranolactone as a new antivirulence lead compound and a chemical tool to increase the knowledge in this field.

## 1. Introduction

The problem of antibiotic resistance, strictly connected to the large overuse of antibiotics in clinical practice and animal husbandry, is rising as a global emergency. There is an urgent need for new drugs to be used as therapeutic agents aimed at virulence factors rather than killing the pathogen, thus providing less selective pressure for evolution of resistance particularly among gram-negative bacteria. It has been reported that half the deaths associated with clinical infection in Europe are associated with multi-drug-resistant bacteria (the so-called “superbugs”) [1]. According to the Infection Disease Society of America (IDSA), the incidence of antibiotic resistance is rapidly increasing. At the same time, fewer new antibiotics are being developed. New compounds that target a variety of virulence factors can serve as adjunctive therapy and potentiate the effect of existing antibiotics. Novel scaffolds in drug discovery are inspired by natural products (NPs) and, although plants remain the major source, marine organisms have been shown to be a treasure of novel lead compounds for drug research [2,3,4,5,6]. Among others, sponges and associated bacteria are potential rich sources of antibacterial compounds [7,8].

**Figure 1:**
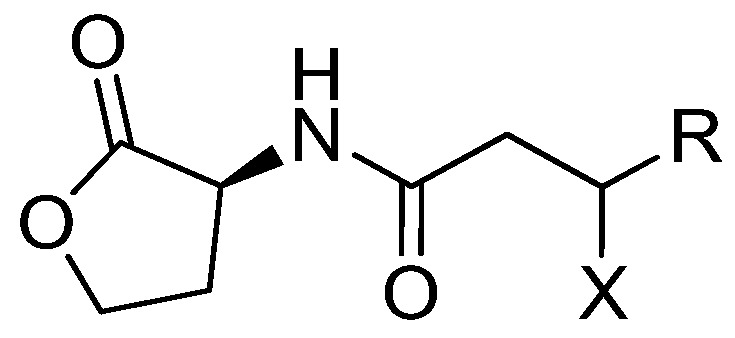
General structure of acyl homoserine lactones (AHLs). R is typically a saturated or monounsaturated C_1_ through C_11_ alkyl chain; X is typically –H, –OH, or =O.

A recent pharmacological approach to control infections relies on studies performed on quorum sensing (QS), a mechanism of cell-cell communication and gene regulation in bacteria. QS acts using chemically different signal molecules, also called autoinducers. Among others, acylated homoserine lactones (AHLs, Figure 1) are used by many Gram-negative bacteria. At low cell density, bacteria produce and release low levels of AHLs, whilst at higher densities, the chemical signals accumulate and once they reach a critical level (quorum) they interact with receptor proteins (LuxR homologs) [9], and induce expression of specific genes, often including those for AHL synthesis (*luxI* homolog). In many pathogens, QS controls the expression of virulence factors, and thus blocking QS will preclude the pathogen from producing those mediators and exotoxins, among others, that accomplish the infection process in the host. One of the best studied models is the opportunistic pathogen *Pseudomonas aeruginosa*, where two AHL-QS systems are organized hierarchically with the RhlI/R system subordinate to the LasI/R one [10,11]. The two QS circuits regulate a range of virulence factors [12,13], that assist *P. aeruginosa* in both acute and chronic infections, mostly in immune-compromised patients [14]. Because QS regulates virulence in pathogens like *P. aeruginosa*, interference with QS (quorum quenching, QQ) has been proposed as a potential therapeutic strategy for fighting pathogens by attenuating the virulence effectors that are regulated by QS [15]. QQ potential was detected in marine sponges and sponge isolates [16,17] and therefore sponges appear as an attractive target to identify novel anti-virulence and QS-inhibiting lead molecules. One means of QS-inactivation is to block the QS signal receptors (e.g., LasR in *P. aeruginosa*) with the antagonist capable of penetrating the bacterial cell, and blocking the cascade of production of virulence factors.

**Figure 2:**
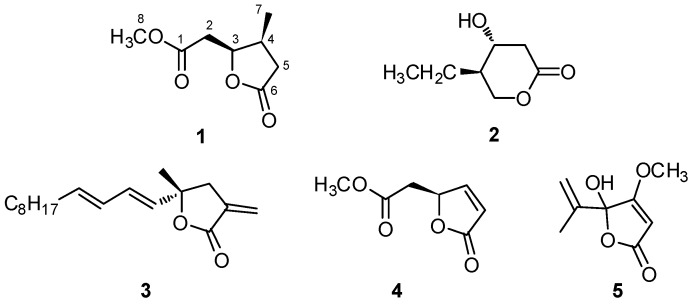
Structures of plakofuranolactone (**1**), three lactones isolated from marine sponges of the genus *Plakortis* (**2**–**4**), and of penicillic acid (**5**).

In a search for molecules active on the QS system, structural analogs of AHLs are obvious candidates. For example, the marine-derived γ-lactones isolated from the red alga *Delisea pulchra* that mimic the lactone moiety of AHLs, have been shown to inhibit AHL-mediated gene expression [18]. Marine sponges of the genus *Plakortis* are a promising source of novel bioactive lactones. The δ-lactone simplactones (e.g., **2**, Figure 2) were isolated in 1999 by Cafieri et al. [19] from a Caribbean specimen of *Plakortis simplex*, the α-methylene-γ-lactone plakolide A (**3**) was isolated by Guanasakera et al. from a Caribbean *Plakortis* sp. [20], and the γ-lactone methyl ester **4** was isolated in 1990 by De Guzman and Schimtz [21] from a specimen of *Plakortis lita* collected in the Pacific Ocean. 

An Indonesian specimen of *Plakortis* cf. *lita*, from which we had previously obtained the hopanoid derivative plakohopanoid [22], was available in our laboratory. Re-examination of the extract of this sponge in the search of novel lactones active as QS inhibitors led to the isolation of the new lactone plakofuranolactone (**1**). In this paper, we report the structural elucidation of the lactone **1** and the evaluation of its QQ activity by in vitro assays.

## 2. Results and Discussion

### 2.1. Isolation and Structural Elucidation of Plakofuranolactone *(**1**)*

An in-depth re-examination of the organic extract of the Indonesian sponge *Plakortis* cf. *lita* was made in the search for novel bioactive lactones. Fraction A2 from the organic extract of the sponge (for extraction/fractionating procedures see ref. [22]) was purified by reversed-phase HPLC on an RP-18 column, followed by normal-phase HPLC on a SiO_2_ column, to yield 1.3 mg of plakofuranolactone (**1**).

The high-resolution ESI mass spectrum of compound **1** showed a pseudomolecular ion peak at *m*/*z* 173.0798 ([M + H]^+^) indicating the molecular formula C_8_H_12_O_4_ (calcd. *m*/*z* 173.0808). The presence of two ester carbonyl signals as the sole *sp*^2^ carbon signals in the ^13^C NMR spectrum, together with the three unsaturations indicated by the molecular formula, suggested a cyclic structure. Analysis of the ^1^H NMR and COSY (COrrelation SpectroscopY) spectra (see Table 1) showed the presence in compound **1** of a –CH_2_–CH(O–)–CH(CH_3_)–CH_2_– part structure (C-2 through C-5, C-7) and an O-methyl group (H_3_-8, singlet at δ 3.71). The HMBC (Heteronuclear Multiple Bond Correlation) correlation of H_2_-2 (δ 2.71) and H_3_-8 with C-1 (δ 172.2) defined the presence of a methoxycarbonyl group at C-2, while the HMBC correlations of H-5a (δ 2.79), H-5b (δ 2.24), and H-3(δ 4.96) with C-6 (δ 178.7) were indicative of a γ-lactone. The concomitant presence of two strong NOESY (Nuclear Overhauser Effect Spectroscopy) correlation peaks between H_3_-7 and H_2_-2 and between H-3 and H-4 was indicative of the *cis* relationship of the two substituents on the γ-lactone ring.

The close structural similarity of compound **1** with the methyl ester **4** isolated from the same *Plakortis* species [21] suggested a common configuration at C-3, and thus the (3*S*,4*S*) absolute configuration for **1**. While in the original report [23] assignment of the absolute configuration of compound **5** was based on its electronic circular dichroism (ECD) spectrum, this approach was not possible for compound **1**, lacking the conjugated π system. It has been recently shown [24] that quantum-mechanical calculation of optical rotation may be a reliable method to predict experimental optical rotations, provided that the conformational behavior of the molecule under investigation is properly considered. Therefore, we used this method to elucidate the absolute configuration of compound **1**. Briefly (see the Experimental Section and Table S1 in Supplementary Materials for details), twelve conformers of (3*S*,4*S*)-**1** in an energy range of 4 kcal/mol were obtained from a 10 ps molecular dynamics simulation in the CVFF (Consistent Valence Force Field) force field. The geometry of each conformer was quantum-mechanically optimized using the program Gaussian 09 [25] at the CAM-B3LYP/AUG-cc-pVDZ level of theory. Optical rotation of each optimized conformer was then calculated using time-dependent density functional theory (TDDFT) at the same theory level. The Boltzmann-weighted mean of the optical rotations of the individual conformers provided a predicted value of –82.8 for the optical rotation of **1**, in excellent agreement with the experimental value of ${\left[\alpha \right]}_{D}^{25}$ = −84. Therefore, the structure of plakofuranolactone (**1**) was defined as 1-methyl (3*S*,4*S*)-3-hydroxy-4-methylhexanedioate 3,6-lactone.

### 2.2. Determination of Non-Inhibitory Concentration (NIC)

**Figure 3:**
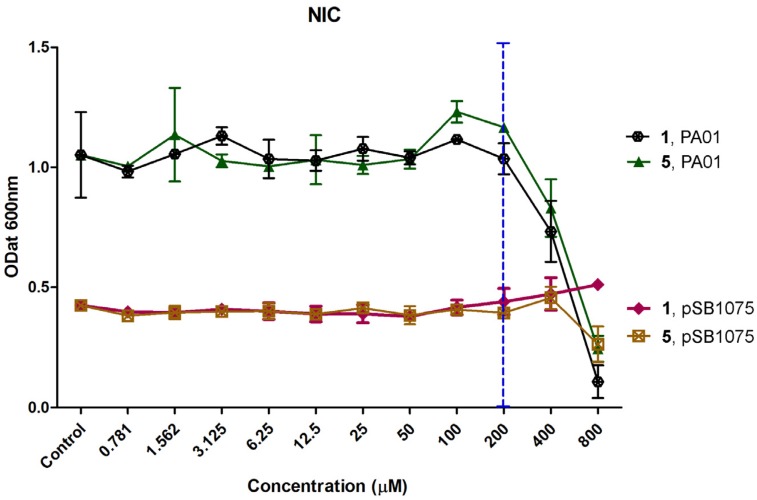
Dose dependent inhibition profile and determination of the non-inhibitory concentration (NIC) of plakofuranolactone (**1**) and penicillic acid (**5**) against *Pseudomonas aeruginosa* PAO1 and *E. coli* pSB1075. The control represents the growth of each tested organism grown in the presence of the diluting solvent used (methanol).

Plakofuranolactone (**1**) was evaluated for determination of its non-inhibitory concentration (NIC) against *E. coli* pSB401 (pSB401), *E. coli* pSB1075 (pSB1075), *P. aeruginosa* PAO1 (PAO1), and *C. violaceum* CV026 (CV026), the four strains used for testing quorum quenching activity. Determination of NIC is important to rule out any possible growth inhibition artifacts during quorum quenching assays. The growth-inhibitory activities of compound **1** and of the positive control **5** were tested at concentrations between 0.781 µM and 800 µM. Compound **1** showed no inhibitory activity against CV026, pSB401, and pSB1075 at any tested concentration, and a dose dependent inhibition against PAO1 above 200 µM. No growth inhibition compared with the control (solvent only) was observed for both compounds and for all the strains at concentrations between 0.781 µM and 200 µM (Figure 3). In addition, the growth curve of the strains was determined in the presence of 200 µM of compound **1**, and no significant effect was found compared to the control (Figure S7). Therefore, this concentration was used for further evaluation of QQ activity.

### 2.3. Quorum Quenching Activity of Plakofuranolactone *(**1**)*

Plakofuranolactone (**1**) shows some structural relationship with an AHL. More interestingly, it appears closely related with penicillic acid (**5**), a fungal metabolite which has been shown to inhibit the QS system of *P. aeruginosa* [26]. We hypothesized that this molecule may interfere with QS activity in *P. aeruginosa* as well, and tested this hypothesis with QS biosensors. Two biosensors, CV026 and pSB401, were used to determine its ability to affect QS systems based on short (C_4_–C_8_) acyl side chain signals. It was found that **1** did not show any inhibitory activity against these biosensors (Figure S8 Supplementary Materials). QQ activity of plakofuranolactone (**1**) was further tested using a long chain (C_10_–C_14_) acyl side chain based reporter assay with the QS biosensor pSB1075. In addition, the influence of compound **1** on the level of two QS-regulated extracellular virulence factors produced by wild-type *Pseudomonas aeruginosa* PAO1 was measured. The fungal metabolite penicillic acid (PA, **5**) was used as a positive control in all the experiments. It was shown that **5** exerts its QQ effect by destabilizing LuxR/LasR, thereby accelerating the proteolytic degradation of this QS regulator protein [27,28].

#### 2.3.1. Dose Dependent Quantification of Bioluminescence for the QQ Assay and Specificity to the LasI/LasR System

**Figure 4:**
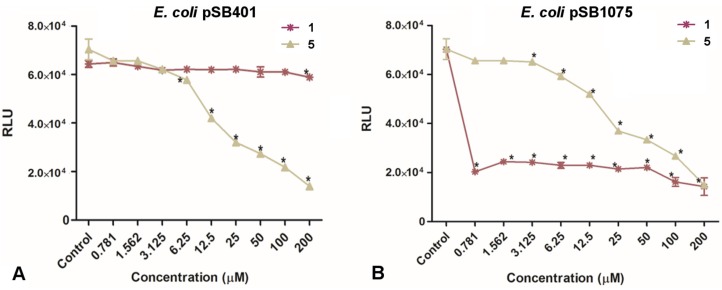
Dose dependent effect of plakofuranolactone (**1**) and penicillic acid (PA, **5**) on the quorum sensing (QS) dependent bioluminescence of the LuxR-based reporter *E. coli* pSB401 induced by 3-oxo-C6-HSL (panel **A**) and the LasR-based reporter *E. coli* pSB1075 induced by 3-oxo-C12-HSL (Panel **B**). Data is expressed as SD of the mean (*n* = 2). Significance (according to Dunnett’s test, *p* < 0.05) is shown with an asterisk.

The reporter strains were used in the presence of the respective cognate AHL signaling molecules, and a decrease in bioluminescence in the presence of the test compound **1** or the control compound **5** was interpreted as QQ activity. Figure 4 shows normalized bioluminescence results for treated (**1** and **5**) reporter strain pSB401 (Figure 4A) and pSB1075 (Figure 4B), activated with their cognate signal molecules after 4 h of incubation.

Compound **1** significantly inhibited the bioluminescence of pSB1075 at all concentrations tested (0.781–200 µM) (ANOVA, Dunnett’s test, *p* < 0.05), while compound **5** showed a dose response for bioluminescence inhibition with IC_50_ at 25 µM (ANOVA, Dunnett’s test, *p* < 0.05). In contrast, there was no inhibition of bioluminescence by pSB401 in the presence of compound **1**. Since both the reporter strains (pSB401 and pSB1075) harbor the same luciferase reporter system and only with the pSB1075 strain was the bioluminescence affected by compound **1**, we suggest that there is no interference with the biochemistry of the bacterial luciferase, and rather compound **1** is specific in its inhibition of the *lasR* system. The pSB1075 biosensor has been previously used for isolation and identification of QQ lead molecules. An example is the isolation of 8-epi-malyngamide C, malyngamide C, malyngolide, and lyngbyoic acid from the extracts of L. majuscula. 8-Epi-malyngamide C and malyngamide C reduced 3-oxo-C12-HSL induced bioluminescence at a concentration of 10 µM [29]. Malyngolide was identified as a QS inhibitor with an EC50 value of 12.2 ± 1.6 µM. This compound was also found to inhibit LasR regulated production of elastase by *P. aeruginosa* PAO1 with EC50 = 10.6 ± 1.8 µM [30]. Lyngbyoic acid also antagonized 3-oxo-C12-HSL induced bioluminescence by pSB1075 with an IC_50_ of approximately 100 µM and reduced pyocyanin and elastase production in *P. aeruginosa* [31]. Further work is required to reveal the mechanism of action by which compound **1** inhibits the Las-QS system.

#### 2.3.2. Inhibition of Virulence Factor Production—Pyocyanin and Total Protease Activity

**Figure 5:**
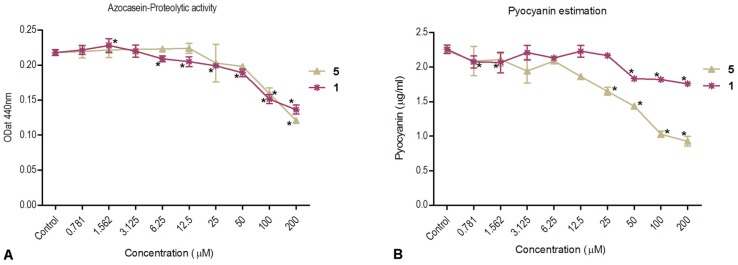
Dose dependent inhibition of proteolytic activity (panel **A**) and pyocyanin production (panel **B**) by plakofuranolactone (**1**) and penicillic acid (**5**). *P. aeruginosa* PAO1 grown in the presence of diluting solvent was used as a negative control in both experiments. Data is expressed as SD of the mean (*n* = 2). Significance (according to Dunnett’s test, *p* < 0.05) is shown with an asterisk.

To study the ability of plakofuranolactone (**1**) to down-regulate QS-regulated virulence factors of *P. aeruginosa*, the levels of two extracellular virulence factors were measured. Virulence factors examined included total protease activity, which is directly controlled by the LasI/R system, and pyocyanin production, which is mainly controlled by the RhlI/R system [32]. *Pseudomonas aeruginosa* PAO1, a wild type opportunistic pathogen strain, was used for these experiments. The activity of protease production was shown to be inhibited by compound **1** in a dose-dependent manner in PAO1. A similar dose dependent inhibitory effect was observed for compound **5** (positive control) (Figure 5A). Accordingly, compound **1** seems to exert its inhibition of protease activity through the inhibition of LasR, in a similar way as compound **5**. However, whilst compound **5** showed also a dose dependent inhibition of pyocyanin production, compound **1** showed a weaker inhibition, and only at the highest concentrations tested. It is frequently found that small molecules targeting the LasR receptor show a remarkably lower IC_50_ in native *P. aeruginosa* than in *E. coli* reporters [33]. Several possible reasons have been proposed, and to some extent demonstrated, for this trend, the most important being the lower membrane permeability and enhanced active efflux in *P. aeruginosa*, and the different levels of receptor expression between *P. aeruginosa* (native levels) and *E. coli* (overexpressed receptor) [34].

## 3. Experimental Section

### 3.1. General Experimental Procedures

High-resolution ESI-MS (ElectroSpray Ionization-Mass Spectrometry) and HR-ESI-HPLC (High Resolution-ElectroSpray Ionization-High Performance Liquid Chromatography) experiments were performed on a Thermo LTQ Orbitrap XL mass spectrometer coupled to a Thermo U3000 HPLC system (Thermo Fisher Scientific Spa, Rodano, Italy). The spectra were recorded by infusion into the ESI source using MeOH as the solvent. NMR spectra were determined on Varian Unity Inova spectrometers (Agilent Technologies, Cernusco sul Naviglio, Italy) at 700 MHz; chemical shifts were referenced to the residual solvent signal (CD_3_OD: δ_H_ 3.31, δ_C_ 49.30). For an accurate measurement of the coupling constants, the one-dimensional ^1^H NMR spectra were transformed at 64-K points (digital resolution: 0.09 Hz). Homonuclear ^1^H connectivities were determined by a COSY experiment. Through-space ^1^H connectivities were evidenced using a NOESY experiment with a mixing time of 500 ms. The HSQC spectra were optimized for ^1^*J*_CH_ = 142 Hz and the HMBC experiments for ^2,3^*J*_CH_ = 8.3 Hz. High performance liquid chromatography (HPLC) was performed on a Varian Prostar 210 apparatus equipped with a Varian 350 refractive index detector (Agilent Technologies, Cernusco sul Naviglio, Italy).

### 3.2. Collection, Extraction, and Isolation

A specimen of *Plakortis* cf. *Lita* (order Homosclerophorida, family Plakinidae) was collected in January 2008 along the coasts of the Bunaken Island in the Bunaken Marine Park of Manado. A voucher sample (No. MAN08-02) has been deposited at the Dipartimento di Farmacia, Università di Napoli Federico II. The sponge (380 mL of volume before extraction and 50 g of dry weight after extraction) was cut into pieces and extracted with MeOH (3 × 1.5 L), MeOH/CHCl_3_ (4 × 1.5 L), and CHCl_3 _(2 × 1.5 L). The MeOH extracts were partitioned with H_2_O and BuOH; the organic layer was added to the CHCl_3 _extracts, affording 8.16 g of dark brown oil, which was chromatographed on a column packed with RP-18 silica-gel. A fraction eluted with H_2_O/MeOH (7:3, 270 mg) was chromatographed on a silica normal-phase column, thus giving a fraction (15.9 mg) which was eluted with 100% AcOET and shown to contain compound **1**. This latter fraction was further subjected to normal-phase HPLC on a SiO_2_ column (Phenomenex, Torrance, CA, USA) [*n*-Hex/AcOET (2:8), Luna Silica, 250 × 4.6 mm, 5 μm], which gave 1.3 mg of pure compound **1**.

Plakofuranolactone (**1**): Colorless amorphous solid, ${\left[\alpha \right]}_{D}^{25}$ = −84 (MeOH); HRESIMS (High Resolution-ElectroSpray Ionization-Mass Spectrometry) (positive ion mode, MeOH) *m*/*z* 173.0798 ([M + H]^+^, C_8_H_13_O_4_^+^ gives 173.0808), *m*/*z* 195.0616 ([M + Na]^+^, C_8_H_12_O_4_Na^+^ gives 195.0628); ^1^H- and ^13^C- NMR: Table 1.

### 3.3. Quantum Mechanical Calculation of the Optical Rotation of Plakofuranolactone *(**1**)*

A conformational search for **1** was performed using molecular dynamics (MD) (Insight II/Discover package [35] and CVFF force field). The MD simulation was performed at 300 K for 10 ns, and the coordinates produced by the simulation were saved every 50 ps, producing 200 structures. Each structure saved from MD was then minimized. The calculation revealed twelve conformers (all within 4 kcal/mol from the lowest energy conformer), differing for the puckering of the five-membered lactone ring and/or for the conformation of the methoxycarbonylmethyl group. Geometry optimizations of each conformer from MD was performed using density functional theory (DFT) with the Gaussian 09 program [25], the CAM-B3LYP functional, the AUG-cc-pVDZ basis set, and the IEF-PCM model for the solvent, MeOH; the results of vibrational frequency analysis confirmed that all conformers were in a true energy minimum, and were used to obtain their free energies. The optical rotation of each DFT-optimized conformer was then calculated using TDDFT at the same theory level (the results are reported in Table S1, Supplementary Materials). Finally, the weighted mean of individual optical rotations was calculated based on DFT relative free energies and Boltzmann statistics (*T* = 298 K).

### 3.4. Determination of Non-Inhibitory Concentration (NIC)

Penicillic acid (**5**) was purchased from Sigma-Aldrich, Rehovot, Israel. Non-inhibitory concentration (NIC) for plakofuranolactone (**1**) and penicillic acid (**5**) was determined by the broth two-fold micro dilution method [35]. Briefly, the compounds were dissolved in methanol to obtain 10 mM stock solutions, which were subsequently serially diluted using Muller-Hinton broth. The inoculum (approximately 5 × 10^5 ^CFU/mL, final concentration) was prepared from an overnight culture and was added (50 µL) to each well containing the compound (200 µL) to give a concentration range of 800–0.781 µM. After incubating 96-well flat bottomed plates aerobically at 37.8 °C for 24 h, the OD (optical density) was measured using a spectrophotometer (600 nm) using a TriStarMultimode Microplate reader (Berthold Technologies GmbH & Co. KG, Bad Wildbad, Germany) to determine NIC values. In addition, the OD was measured at regular intervals during the growth, and growth curves were built from the resulting values. Negative controls (culture + methanol) were included.

### 3.5. Dose Dependent QQ Assay

Well diffusion QQ assay was performed using *C. violaceum* CV026 following a pre-established protocol [36], whereas bioluminescence based QQ assay using *E. coli* pSB401 and *E. coli* pSB1075 reporters were quantified on a TriStar Multimode Microplate reader (Berthold Technologies GmbH & Co. KG) as reported [37]. Plasmid pSB401 was constructed using the *P. luminescens* *luxCDABE* operon controlled by the *PluxI* gene promoter together with the *V. fischeri* *luxR* DNA fragment and when transformed in *E. coli* it emits luminescence in response to the exogenous addition of AHLs with medium (C_6_–C_8_) acyl side chain lengths [37]. LasR-based reporter plasmid pSB1075 contains the *lasR* gene, and the promoter of *lasI^−^* controls the expression for the reporter operon *luxCDABE* and emits luminescence in response to AHLs with long (≥C_10_) acyl side chains [37]. Briefly, the stock solutions (10 mM) of compounds **1** and **5** were serially diluted to NIC concentrations (0.781–200 µM) in Luria-Bertani (LB) medium. The inocula of the reporter strains (OD_600_ 0.01, final concentration) were prepared from an overnight culture and added (50 µL) to each well. The inoculum was supplemented with *N*-(3-oxo-hexanoyl)-l-homoserine lactone (3-oxo-C6-HSL, 1 μM final concentration) and *N*-(3-oxo-dodecanoyl)-l-homoserine lactone (3-oxo-C12-HSL, 2 μM final concentration) in order to stimulate QS of the pSB401 and pSB1075 biosensors, respectively. The bioluminescence was recorded every 30 min for 7 h at 30 °C. The production of bioluminescence in the graphs is given as the relative light units (RLU), obtained at 4 h.

### 3.6. Inhibition of Virulence Factor Production—Pyocyanin and Protease

Inhibitory activity of pyocyanin production by *Pseudomonas aeruginosa* PAO1 was performed as described earlier [38]. Briefly, an overnight culture of *P. aeruginosa* PAO1 was diluted with LB medium to an OD_600_ of 0.2 and 4.5 mL were transferred to a 20 mL test tube. The diluted cultures were supplemented with 250 µL of test compounds plakofuranolactone (**1**) or penicillic acid (**5**). Methanol (solvent in which test compounds were dissolved) was used for negative controls. After overnight incubation at 37 °C and 200 rpm, 3 mL of chloroform was added to each test tube and mixed vigorously. The organic layer was collected by centrifugation (2000 *g*) and transferred to a fresh tube. Hydrochloric acid (0.2 M, 1 mL) was added to the organic layer and the absorbance was measured at 520 nm. The experiments were performed in triplicate. Pyocyanin concentration (µL/mL) was calculated as:

P=(OD×17.072)×1.5

where OD is optical density value obtained at 520 nm, 17.072 is the extinction coefficient used to obtain the value in µg/mL, and 1.5 is the dilution factor (3 mL from initial 4.5 mL of chloroform was used) [39].

Proteolytic activity was determined using the azocasein assay as previously described [40]. Briefly, we treated wild type *P. aeruginosa* PAO1 strains with test compounds **1** and **5** in a dose dependent manner at NIC concentration. An overnight inoculum (approximately 5 × 10^5 ^CFU/mL, final concentration) was prepared and was added to each well containing test compounds **1** or **5** (0.781–200 µM) and was incubated at 37 °C for 16 h. Once the OD_600_ reached 0.4, the plate was centrifuged and 30 µL of supernatant was transferred to a clean Eppendoff tube containing 50 µL of 0.8% azocasein. The mixture was incubated at 37 °C for 12 h. Subsequently, the mixture was incubated at room temperature for 15 min with 240 µL of 10% (*w*/*v*) trichloroacetic acid (TCA), and centrifuged for 10 min at 15,000× *g*. Prior to spectroscopic measurement at OD_440_, 240 μL of 1 M NaOH was added. *P. aeruginosa* PAO1 without any treatment in diluting solvent (methanol and sterile culture media) was used as the negative control. 

### 3.7. Statistical Analysis

The significance differences between the mean values of tested subjects from their corresponding controls were tested using ANOVA Dunnett’s test (*p* < 0.05) using GraphPad Prism software version 5.01. All the assays were performed in duplicates.

## 4. Conclusions

Quorum quenching, a novel approach which is based on the inhibition of the QS systems that regulate cell-cell communication and the production of virulence factors in bacteria, is now regarded as a potential therapeutic strategy to design novel drugs that will reduce virulence factors while not acting as bactericidals. However, it should be noted that the potential for the emergence of pathogen resistance against antivirulence drugs is still under debate [41,42,43].

A novel γ-lactone, plakofuranolactone (**1**), which shows a strong QQ activity at submicromolar concentration, has been discovered from the extract of the Indonesian sponge *Plakortis* cf. *lita*. The QQ activity of compound **1** was demonstrated using a biosensor, the QS-reporter strain pSB1075, in which a reporter gene (bioluminescence) is under control of the LasI/R system. The experiments showed that compound **1** can effectively inhibit AHL-induced bioluminescence. In addition, in an experiment on a wild-type strain of *P. aeruginosa*, the total protease activity (one of the virulence factors of *P. aeruginosa* which is under control of the LasI/R system) was decreased by treatment with compound **1**. Therefore, plakofuranolactone can be regarded as a model scaffold to design a first generation of antivirulence drugs. The lack of QQ activity against short-chain AHL-QS systems (based on the results with biosensors *C. violaceum* CV026 and *E. coli* pSB401) suggests that the QQ activity of compound **1** is not general, but rather specific to certain QS systems. Furthermore, it supports the fact that the reduction in bioluminescence observed when compound **1** was added in the pSB1075 assay was a result of QQ and not a direct inhibition of bioluminescence, given that pSB401 is also a bioluminescence-based biosensor.

In a study on cellular localization of secondary metabolites in a *Plakortis* sponge [44], it has been shown that all the secondary metabolites found in the extract of the sponge are mainly or exclusively present in the bacterial fraction, and are therefore of likely microbial origin. It is even more likely that plakofuranolactone (**1**), a metabolite that is involved in bacterial cell-to-cell communication, is a product of bacterial metabolism, although no positive evidence is presently available in this respect. Further studies to discover the biosynthetic genes for plakofuranolactone and identify their host organism are in progress.

## Figures and Tables

**Table 1:** NMR data of plakofuranolactone (**1**) (700 MHz, CD_3_OD).

Pos.		δ_H_ [mult., *J* (Hz)]	δ_C_ [mult.]	COSY	HMBC
1			172.2 (C)		
2		2.71 (d, 7.1)	35.7 (CH_2_)	3, 8	1, 3, 4
3		4.96 (q, 6.7)	81.0 (CH)	2, 4, 5a	1, 2, 4, 5, 6, 7
4		2.75 (m)	33.8 (CH)	3, 5a, 5b, 7	5, 6, 7
5	a	2.79 (dd, 16.9, 8.0)	37.5 (CH_2_)	3, 4, 5b, 7	3, 4, 6, 7
	b	2.24 (dd, 16.9, 4.4)		4, 5a, 7	3, 4, 6, 7
6			178.7 (C)		
7		1.01 (d, 7.1)	14.0 (CH_3_)	4, 5a, 5b	3, 4, 5
8		3.71 (s)	52.3 (CH_3_)	2	1

## Supplementary Materials

The following are available online at www.mdpi.com/1660-3397/15/3/59/s1. Table S1: Calculated optical rotations for individual conformers of **1**, Figure S1: ^1^H-NMR spectrum of compound **1** (700 MHz, CD_3_OD), Figure S2: Expansion of the ^1^H-NMR spectrum of compound **1**, Figure S3: COSY spectrum of compound **1**, Figure S4: NOESY spectrum of compound **1**, Figure S5: HSQC spectrum of compound **1**, Figure S6: HMBC spectrum of compound **1**, Figure S7: Growth curve inhibition profile of plakofuranolactone (**1**) against *Pseudomonas aeruginosa* PAO1, *E. coli* pSB401 and *E. coli* pSB1075, Figure S8: Effects of compounds **1** and **5** on *C. violaceum* CV026.
