# Supplementary material for: Plakofuranolactone as a Quorum Quenching Agent from the Indonesian Sponge Plakortis cf. lita
Source: Mar Drugs. 2017 Feb 28;15(3):59. doi: 10.3390/md15030059 (PMC5367016; doi:10.3390/md15030059)
Supplement: Supplementary file 1 [file marinedrugs-15-00059-s001.pdf]

Supplementary Materials: Plakofuranolactone as a Quorum Quenching Agent from the Indonesian Sponge *Plakortis* cf. *lita*

Valeria Costantino, Gerardo Della Sala, Kumar Saurav, Roberta Teta, Rinat Bar-Shalom, Alfonso Mangoniand Laura Steindler

Table S1. Calculated relative energies (ΔE), relative free energies (ΔG), and optical rotations of the conformers of **1** at the CAM-B3LYP/AUG-cc-pVDZ level.

Conformer	ΔE (kcal/mol)	ΔG (kcal/mol)	Boltzmann factor[a]	[α]
1	0.00	0.00	1.00	–120.1
2	0.35	0.37	0.54	–130.5
3	0.45	0.49	0.43	+5.4
4	0.61	0.48	0.45	-82.9
5	0.73	0.83	0.25	–84.4
6	1.02	1.19	0.13	+52.5
7	1.22	1.15	0.14	–55.9
8	1.65	2.06	0.03	–11.6
9	1.89	2.27	0.02	–66.8
10	3.03	3.58	0.00	+65.8
11	3.72	4.08	0.00	+59.6
12	3.91	4.49	0.00	–32.7
		**weighted mean**		**–82.8**
		**experimental value**		**–84.0**

[a] At T = 298 K, calculated on ΔG.

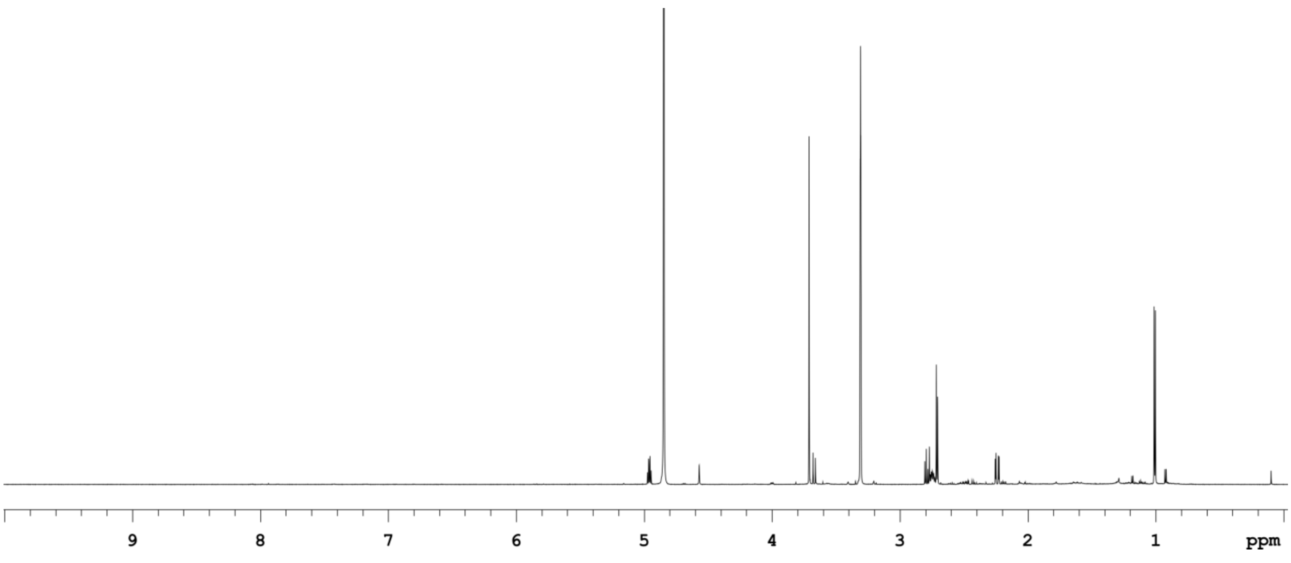

Figure S1. ^{1}H-NMR spectrum of plakofuranolactone (**1**) (700 MHz, CD_3OD).

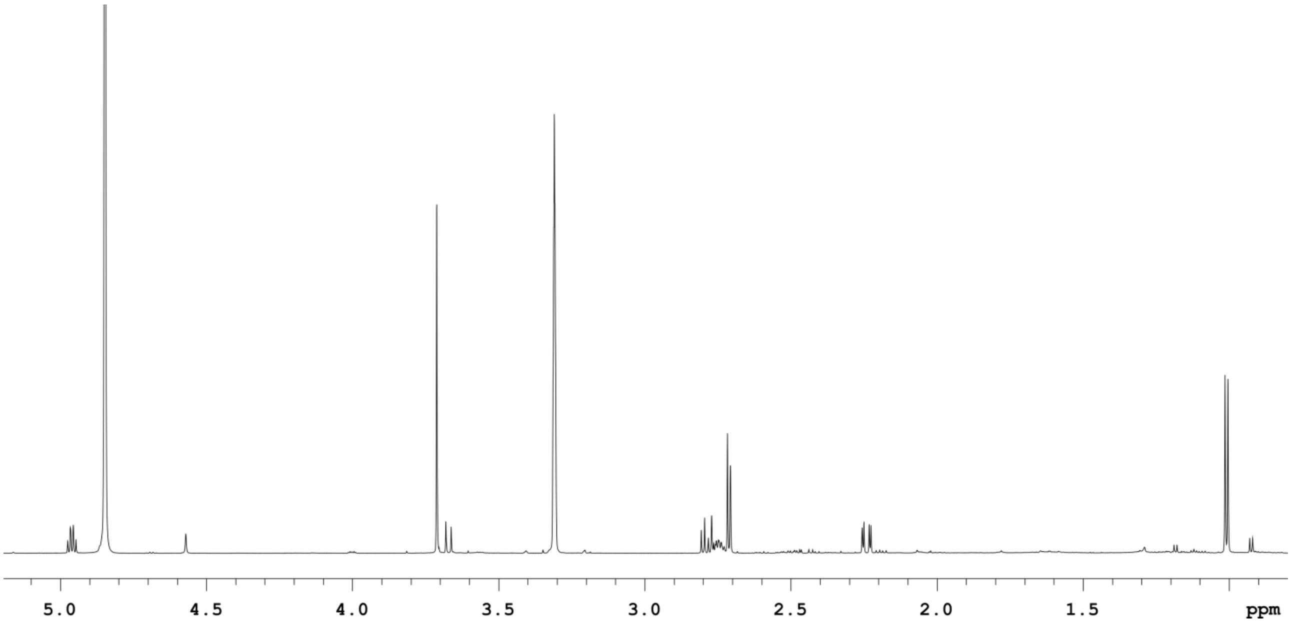

Figure S2. Expansion of the ^{1}H-NMR spectrum of plakofuranolactone (**1**) (700 MHz, CD_3OD).

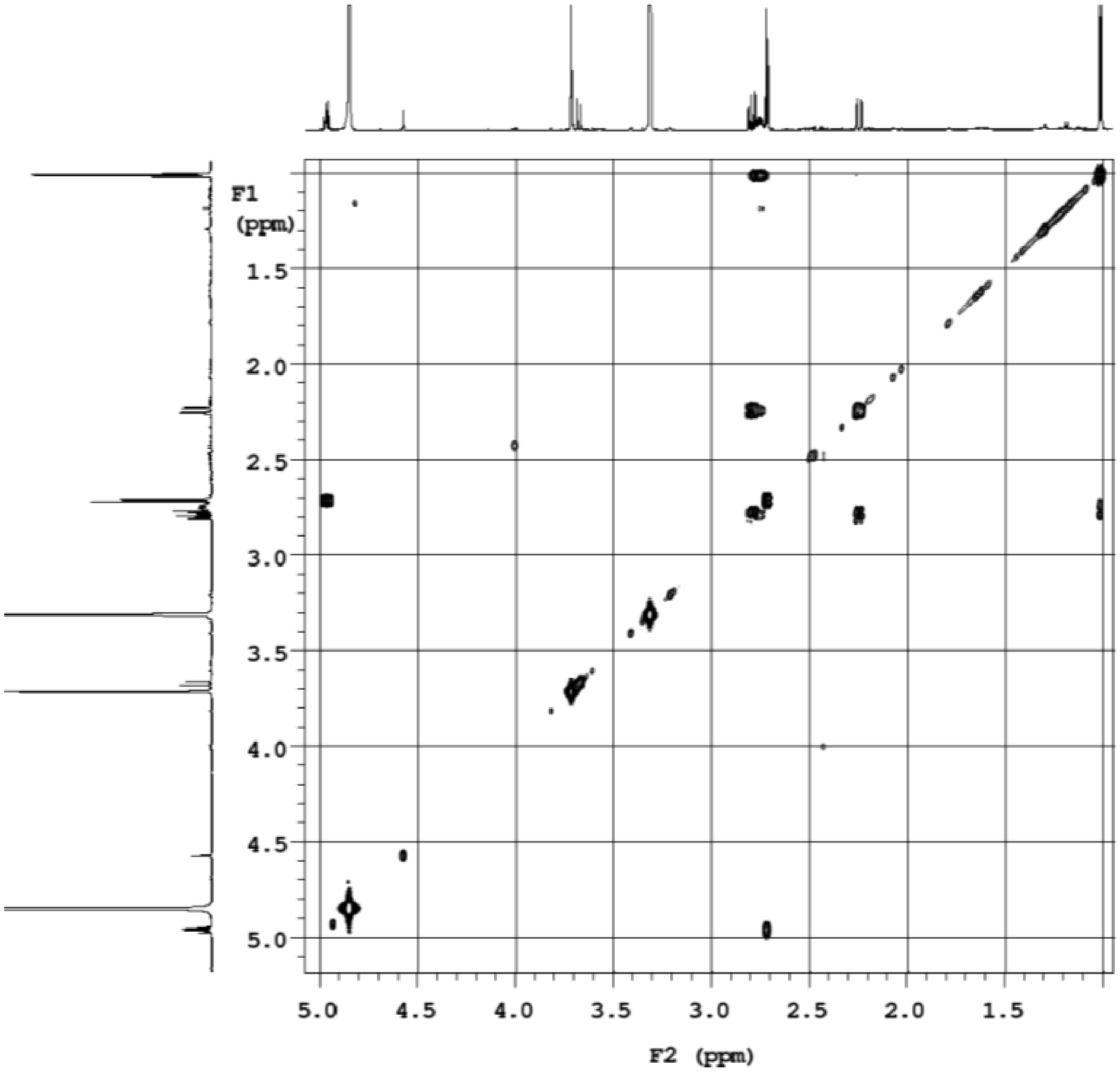

Figure S3. COSY spectrum of plakofuranolactone (**1**) (700 MHz, CD_3OD).

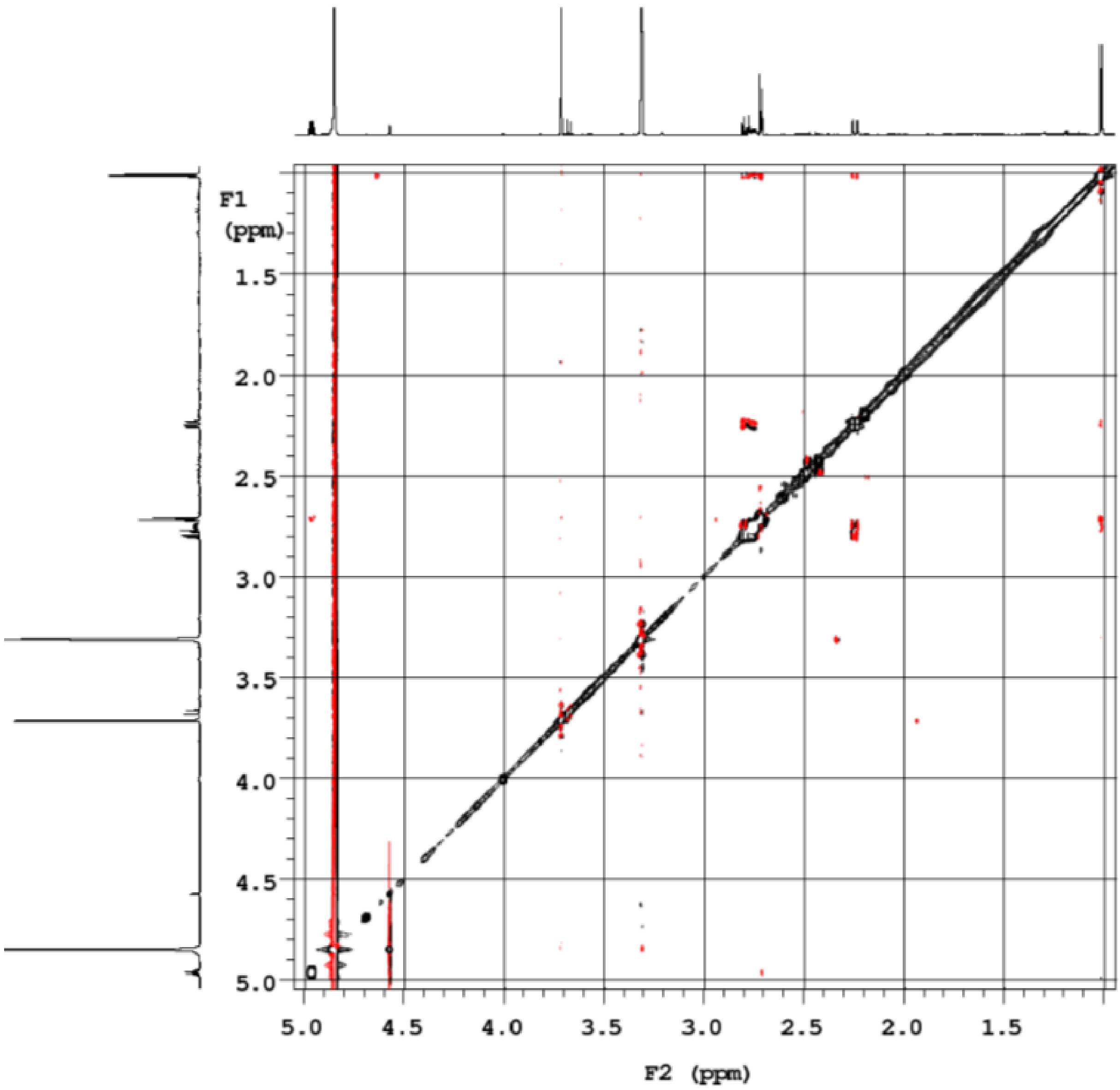

Figure S4. NOESY spectrum of plakofuranolactone (**1**) (700 MHz, CD_3OD).

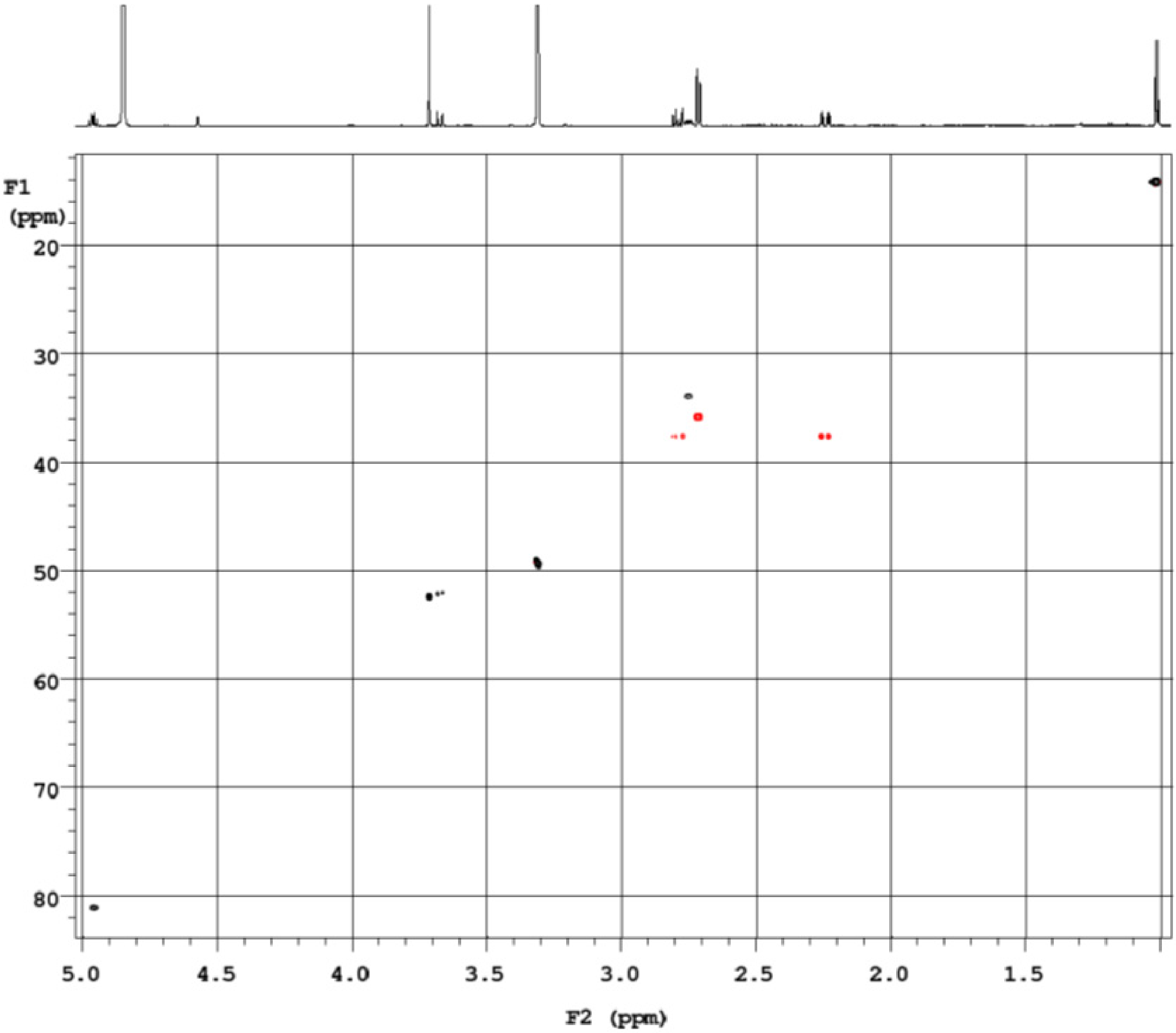

Figure S5. HSQC spectrum of plakofuranolactone (**1**) (700 MHz, CD_3OD).

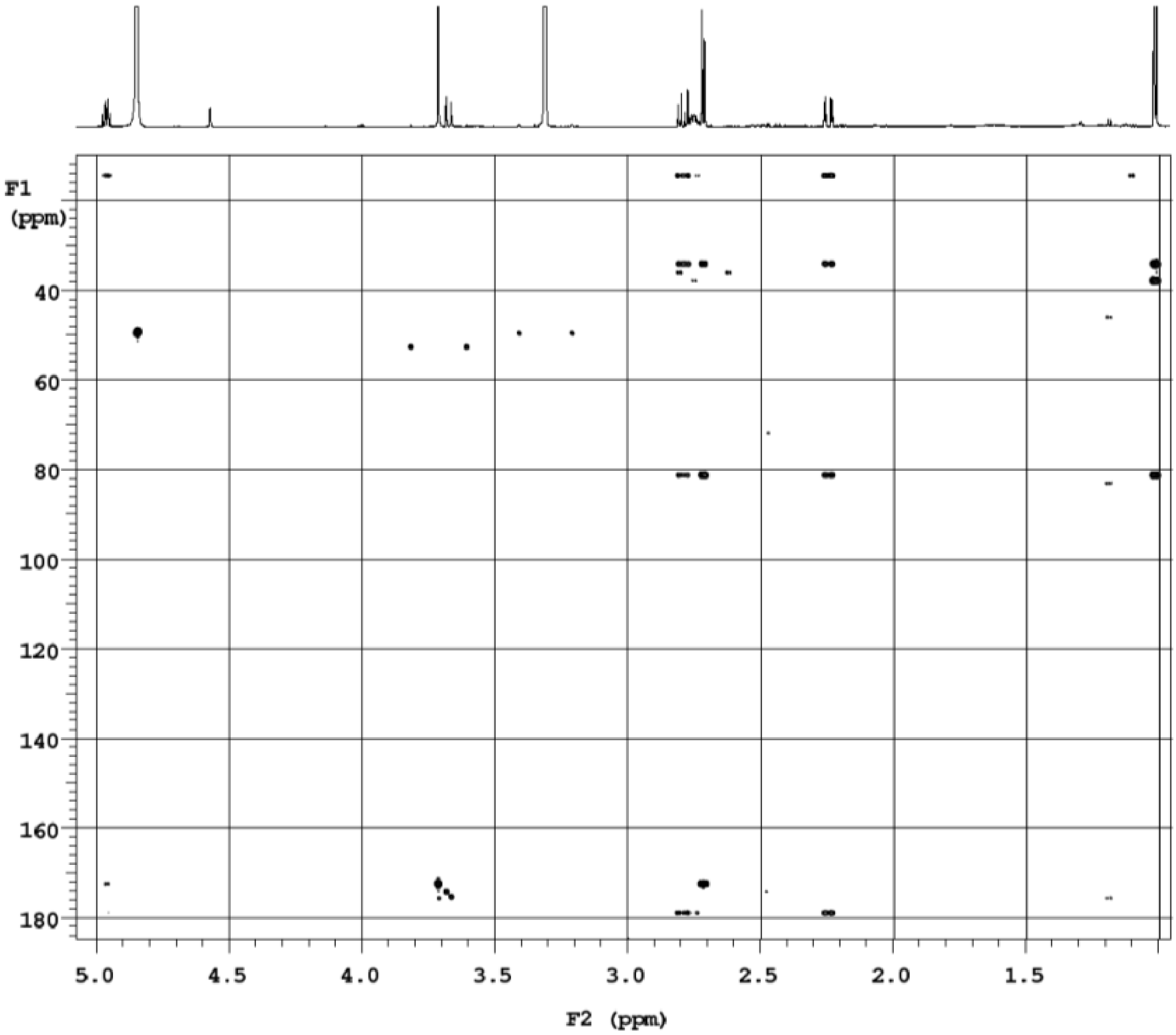

Figure S6. HMBC spectrum of plakofuranolactone (**1**) (700 MHz, CD_3OD).

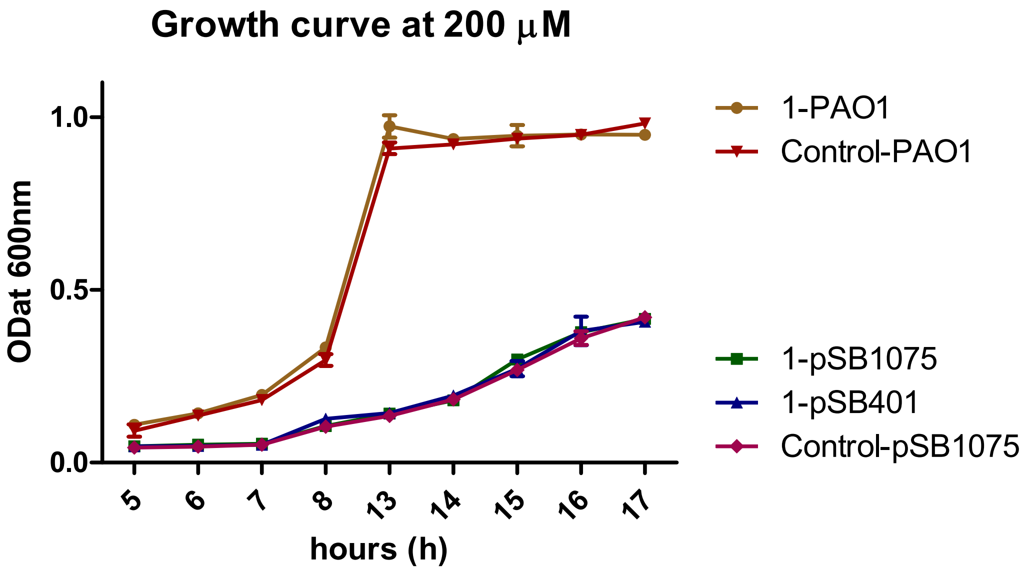

Figure S7. Growth curve inhibition profile of plakofuranolactone (**1**) at 200 µM against *Pseudomonas aeruginosa* PAO1, *E.coli* pSB401 and *E.coli* pSB1075. The control represents the growth of the tested organism grown in the presence of the solvent used for dilutions (methanol).

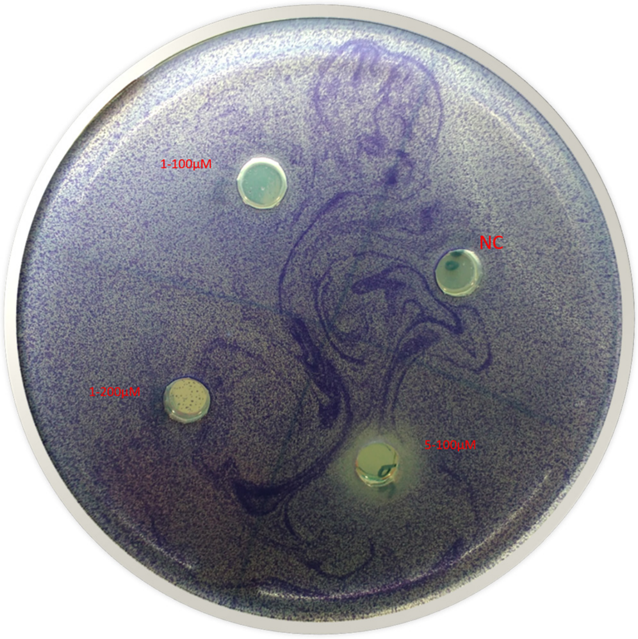

Figure S8. Overlaid well diffusion assay of plakofuranolactone (**1**) and penicillic acid (PA, **5**) on the QS dependent CviI/CviR-based reporter *Chromobacterium violaceum* strain CV026 induced by 3-oxo-C6-HSL (final concentration of 1 µM). The solvent used for dilution was used as a negative control (NC). A white halo around the well, representing the inhibition of violacein production, interpreted as QQ activity, can only be observed in the treatment PA, **5**.
